# Perceived Chronic Traumatic Encephalopathy and Suicidality in Former Professional Football Players

**DOI:** 10.1001/jamaneurol.2024.3083

**Published:** 2024-09-23

**Authors:** Rachel Grashow, Douglas P. Terry, Grant L. Iverson, Heather DiGregorio, Inana Dairi, Cheyenne Brown, Paula S. Atkeson, Alicia J. Whittington, LeRoy Reese, Jonathan H. Kim, Niki Konstantinides, Herman A. Taylor, Frank E. Speizer, Daniel H. Daneshvar, Ross D. Zafonte, Marc G. Weisskopf, Aaron L. Baggish

**Affiliations:** 1 Football Players Health Study at Harvard University, Harvard Medical School, Boston, Massachusetts; 2 Department of Environmental Health, Harvard T.H. Chan School of Public Health, Boston, Massachusetts; 3 Vanderbilt Sports Concussion Center, Department of Neurological Surgery, Vanderbilt University Medical Center, Nashville, Tennessee; 4 Department of Physical Medicine and Rehabilitation, Spaulding Rehabilitation Hospital, Charlestown, Massachusetts; 5 Department of Physical Medicine and Rehabilitation, Harvard Medical School, Charlestown, Massachusetts; 6 Department of Physical Medicine and Rehabilitation, Schoen Adams Research Institute at Spaulding Rehabilitation, Charlestown, Massachusetts; 7 Sports Concussion Program, Mass General for Children, Boston, Massachusetts; 8 Prevention Research Center, Morehouse School of Medicine, Atlanta, Georgia; 9 Emory Clinical Cardiovascular Research Institute, Emory University School of Medicine, Atlanta, Georgia; 10 Cardiovascular Research Institute, Morehouse School of Medicine, Atlanta, Georgia; 11 Channing Division of Network Medicine, Brigham and Women’s Hospital, Harvard Medical School, Boston, Massachusetts; 12 Cardiovascular Performance Program, Massachusetts General Hospital, Boston; 13 Department of Cardiology, Lausanne University Hospital (CHUV) and Institute for Sport Science, University of Lausanne (ISSUL), Lausanne, Switzerland

## Abstract

**Importance:**

Participation in American-style football (ASF) has been linked to chronic traumatic encephalopathy neuropathological change (CTE-NC), a specific neuropathologic finding that can only be established at autopsy. Despite being a postmortem diagnosis, living former ASF players may perceive themselves to have CTE-NC. At present, the proportion and clinical correlates of living former professional ASF athletes with perceived CTE who report suicidality are unknown.

**Objective:**

To determine the proportion, clinical correlates, and suicidality of living former professional ASF players with perceived CTE.

**Design, Setting, and Participants:**

A cross-sectional study within the Football Players Health Study at Harvard University was conducted from 2017 to 2020. Using electronic and paper surveys, this population-based study included former ASF players who contracted with a professional league from 1960 to 2020 and volunteered to fill out a baseline survey. Data for this study were analyzed from June 2023 through March 2024.

**Exposures:**

Data included demographics, football-related exposures (eg, position, career duration), and current health problems (anxiety, attention-deficit/hyperactivity disorder, depression, diabetes, emotional and behavioral dyscontrol symptoms, headache, hyperlipidemia, hypertension, low testosterone level, pain, sleep apnea, and subjective cognitive function).

**Main Outcomes and Measures:**

The proportion of participants reporting perceived CTE. Univariable and multivariable models were used to determine clinical and suicidality correlates of perceived CTE.

**Results:**

Among 4180 former professional ASF players who volunteered to fill out a baseline survey, 1980 (47.4%) provided follow-up data (mean [SD] age, 57.7 [13.9] years). A total of 681 participants (34.4%) reported perceived CTE. Subjective cognitive difficulties, low testosterone level, headache, concussion signs and symptoms accrued during playing years, depressive/emotional and behavioral dyscontrol symptoms, pain, and younger age were significantly associated with perceived CTE. Suicidality was reported by 171 of 681 participants with perceived CTE (25.4%) and 64 of 1299 without perceived CTE (5.0%). After adjusting for established suicidality predictors (eg, depression), men with perceived CTE remained twice as likely to report suicidality (odds ratio, 2.06; 95% CI, 1.36-3.12; *P* < .001).

**Conclusions and Relevance:**

This study found that approximately one-third of living former professional ASF players reported perceived CTE. Men with perceived CTE had an increased prevalence of suicidality and were more likely to have health problems associated with cognitive impairment compared with men without perceived CTE. Perceived CTE represents a novel risk factor for suicidality and, if present, should motivate the diagnostic assessment and treatment of medical and behavioral conditions that may be misattributed to CTE-NC.

## Key Points

**Question:**

What is the proportion and clinical profile of living former American-style football (ASF) players who perceive themselves to have chronic traumatic encephalopathy (CTE)?

**Findings:**

This cross-sectional study found that approximately 34% of former professional ASF players reported perceived CTE. Perceived CTE was significantly associated with a number of health problems, and more men with perceived CTE reported suicidality compared with those without.

**Meaning:**

Perceived CTE is common among former professional ASF players and is associated with discrete clinical features, including suicidality.

## Teaser

This cross-sectional study gathered data using surveys from former professional players of American-style football to gauge how many perceive that they have chronic traumatic encephalopathy and determine clinically relevant correlates, including suicidality.

## Introduction

Chronic traumatic encephalopathy neuropathological change (CTE-NC), the autopsy-based finding of phosphorylated tau aggregates in neurons at the depth of cortical sulci around a small vessel,^1^ has been reported at high rates among deceased former professional American-style football (ASF) players (Box).^2,3^ Recent work has attempted to delineate clinical profiles of living men subsequently shown to have CTE-NC.^4^ Consensus criteria for traumatic encephalopathy syndrome (TES),^5^ the proposed antemortem clinical disorder associated with CTE-NC, include cognitive impairment and neurobehavioral dysregulation not fully accounted for by alternative neurologic, psychiatric, or medical conditions (Box). However, the extent to which CTE-NC is the underlying cause of premortem neuropsychiatric problems remains uncertain,^1^ as there is no diagnostic test that can accurately confirm or exclude CTE-NC in living people. Furthermore, clinicopathological correlation studies report low specificity of prior iterations of TES criteria for autopsy-confirmed CTE-NC.^4,6^ Nonetheless, ASF players, lay people, clinicians, and popular media outlets^7,8,9,10,11,12,13^ often refer to CTE as a singular entity occurring in living and deceased individuals to represent CTE-NC and/or TES interchangeably (Box). Used in these contexts, CTE is often presented as manifesting in living people as cognitive impairment, mood disorders, and suicidality, even though depression and suicidality are not part of the TES core clinical features.^5^

Box. **Key Terms and Definitions**

Chronic traumatic encephalopathy-neuropathological change (CTE-NC): a postmortem neuropathological entity defined by tau protein deposition patterns identified on autopsy.
Traumatic encephalopathy syndrome (TES): a possible in vivo clinical syndrome associated with CTE-NC identified in populations exposed to repetitive head injury. Core features of TES include cognitive impairment, neurobehavioral dysregulation, or both.
Chronic traumatic encephalopathy (CTE): the term often used in the literature and by lay people to refer to CTE-NC and a broad range of psychiatric and neurological clinical signs and symptoms that were present during life in the brain donors. Participants in this study were asked if they believed they had CTE.
Perceived CTE: a term denoting the perception of living individuals that they have what they consider to be CTE, CTE-NC, or clinical manifestations of CTE-NC.

Neither CTE-NC nor TES is curable or has established treatments. Accordingly, a person’s perception that they have or are simply at increased risk for CTE may have similar implications to diagnoses of progressive incurable neurologic diseases. For example, suicide rates are increased among people with Alzheimer disease,^14^ Parkinson disease,^15,16,17,18^ Huntington disease,^19,20,21^ and amyotrophic lateral sclerosis,^22,23,24^ although it is unknown how underlying organic changes and psychological stress differentially contribute to suicidality in these populations.^25^ At present, the extent to which living former professional ASF players perceive that they have CTE-NC is unknown. Similarly, associations between perceived CTE and demographics, football exposures, other health conditions, and suicidality have not been rigorously established.

This study had 2 objectives. First, we sought to determine the proportion of living former professional ASF players with perceived CTE. Second, we aimed to identify the clinically relevant correlates, including suicidality, of perceived CTE. To accomplish these aims, we compared characteristics of living former professional ASF players with and without perceived CTE using data from the Football Players Health Study at Harvard University.^26^

## Methods

### Study Participants

The Football Players Health Study^26^ initiated a longitudinal cohort of former professional ASF players in 2015. Players who contracted with any professional football league after 1960, when hard plastic helmets were largely adopted,^27^ were eligible. Electronic and paper invitations were used to invite 15 011 participants, of whom 4180 (27.2%) enrolled as of October 2019. Participants resemble those who did not enroll in age, position, and current body mass index (BMI).^26^ In the first survey, demographic, football, and health data were collected, and 1980 players (47.4%) provided additional data on perceptions of CTE and self-harm. Data for this study were analyzed from June 2023 through March 2024. The institutional review board of the Harvard T.H. Chan School of Public Health approved this study. Participants provided informed consent prior to enrollment.

### Demographics and Football Exposures

Age was determined using dates of birth and survey completion. Participants chose the category that best described their race and ethnicity, which were then categorized into Black, Native Hawaiian/Pacific Islander/Asian/American Indian/Alaskan Native, White, and missing.^28^ Participants self-reported ASF career duration, age at first football exposure, and position (classified as linemen [offensive line, defensive line, or linebacker] or nonlinemen [all other positions]). BMI at the time of professional play was calculated from self-reported weight and height. Sports-related head injury accrued during years of active play was measured using a concussion signs and symptom (CSS) score^28,29,30,31^ and total number of episodes of loss of consciousness as previously described (eMethods in Supplement 1).^32,33,34,35,36^ Participants were asked, “During your active playing years, did you take any medications or other drugs to help performance?” and the response options were yes or no.

### Clinical Variables

To assess perceived CTE, participants were asked, “Do you believe you have chronic traumatic encephalopathy (CTE)?” and offered the options yes or no. Deliberately, no definition of CTE was provided. It is not possible to know whether participants conceptualized CTE as a specific form of neuropathology, a psychiatric condition, a neurological disease, a neurodegenerative disease, some combination of these, or something else. As described previously,^37^ we ascertained health conditions known to be associated with cognitive dysfunction or common in former athletes (eMethods in Supplement 1). Depression symptoms over the past 2 weeks were assessed using the 9-item Patient Health Questionnaire (PHQ-9).^38^ In the full version, the final item assesses suicidal ideation (ie, “Thoughts that you would be better off dead or hurting yourself in some way”). If the respondent endorsed several days, more than half the days, or nearly every day for this item, he was classified as experiencing suicidality. Some analyses used all 9 items while others excluded the suicidal ideation question (PHQ-8).^39^ Anxiety symptoms over the past 2 weeks were assessed using the 7-item Generalized Anxiety Disorder (GAD-7) questionnaire (eMethods in Supplement 1).^40^ Both the PHQ-9 and the GAD-7 response options include none, several days, more than half the days, and nearly every day.

Perceived cognitive difficulties were assessed using the Quality of Life in Neurological Disorders (Neuro-QoL) Applied Cognition General Concerns scale, which queries 8 cognitive symptoms over the previous week.^41,42^ Emotional and neurobehavioral dyscontrol was measured using the Neuro-QoL Emotional and Behavioral Dyscontrol questionnaire,^41^ which queries the frequency of symptoms (eg, being easily upset, losing one’s temper, being easily irritated) over the prior week. Both Neuro-QoL instruments produced raw scores that were converted into normative T-scores (mean [SD] 50 [10]) based on US general population norms. Traditionally, low scores represent more cognitive difficulties. Given that all other scales in this study associate worse functioning with higher scores, Neuro-QoL Cognition T-scores were assigned as negative values such that higher scores reflected more cognitive difficulties.

Pain intensity was assessed with the Brief Pain Inventory^43^ survey item “Please rate your pain by marking the oval with the number that best describes your pain on average.” Zero represented no pain, and 10 represented pain as bad as the participant could imagine. Habitual physical activity was dichotomized into those who fulfilled guidelines for weekly physical activity (ie, >149 minutes of moderate exercise [walking or jogging] or >74 minutes of vigorous exercise [running or other aerobic activity]) vs those who met neither criterion.^44^

### Statistical Analysis

Bivariate associations were calculated between perceived CTE status and demographic, football-related, and current health variables using Kruskal-Wallis rank sum and χ^2^ tests. We used logistic regression to estimate odds ratios (ORs) of reporting perceived CTE after adjusting for demographic, football-related, and current health. We ran separate logistic regression models to predict suicidality, defined by the presence of suicidal ideation at least several days over the past 2 weeks, using the PHQ-9 suicidality question as the dependent variable. In adjusted models, players who responded “not at all” served as the reference group, compared with those who reported suicidality for several days or more. The PHQ-8 was used to examine associations between depressive symptom severity and suicidality. All models were adjusted for race and ethnicity, age, BMI, age of first football exposure, career duration, linemen status, use of performance-enhancing drugs, CSS score, ADHD, anxiety, depression, diabetes, headaches, heart conditions, hypertension, hyperlipidemia, low testosterone level, sleep apnea, and stroke. We stratified by perceived CTE status to compare adjusted associations with suicidality across perceptions of CTE. As a sensitivity model, each logistic regression model was run with additional dementia-related variables, including self-reported diagnoses of Alzheimer dementia, vascular dementia, or “other” dementia. We used multinomial logistic regression to estimate odds ratios by multiple levels of frequency of suicidality. Additional sensitivity analysis used inverse probability weighting for dropout^45^ to reduce potential bias resulting from nonrandom follow-up queries of perceived CTE (eMethods in Supplement 1). Statistical significance was considered at a 95% confidence, and analyses were conducted using R Language for Statistical Computing version 4.3 (R Foundation).^27^

## Results

The 1980 participants who responded to the PHQ-9 and CTE perception questions on a secondary survey resembled the full cohort of 4189 (eTable 1 in Supplement 1). Demographic factors, football-related exposures, and health conditions among men with and without perceived CTE are shown in the Table. Respondents (mean [SD] age, 57.7 [13.9] years at the time of survey completion) had a mean (SD) professional football career duration of 7 (4) years. One in 3 former players self-identified as Black (595/1908; 30.1%). A total of 681 of 1980 men (34.4%) reported perceived CTE. Former players reporting perceived CTE were a mean of 5.4 years younger than men without perceived CTE. Compared with men without perceived CTE, men with perceived CTE had higher CSS scores, greater use of performance-enhancing drugs, worse depression and anxiety symptoms, and higher proportions of prescription pain medication use, sleep apnea, and low testosterone level. The proportions of these conditions were similar among those who provided data on 1 or both occasions (eTable 1 in Supplement 1).

**Table.** Cohort Characteristics by Perceived CTE Status

Characteristic	No. (%)			*P* value
	Total (N = 1980)	No perceived CTE (n = 1299)	Perceived CTE (n = 681)	
Age, mean (SD), y	57.7 (13.9)	59.5 (14.2)	54.1 (12.7)	<.001
Race and ethnicity^a^				
Black or African American	595 (30.1)	347 (26.7)	248 (36.4)	<.001
Native Hawaiian/Pacific Islander/Asian/American Indian/Alaskan Native	58 (2.9)	31 (2.4)	27 (4.0)	
White	1303 (65.8)	904 (69.6)	399 (58.6)	
Missing data, No.	24 (1.2)	17 (1.3)	7 (1.0)	
Body mass index, mean (SD)^b^	30.8 (4.91)	30.4 (4.61)	31.6 (5.4)	<.001
Missing data, No.	14	8	6	
Meets physical activity standards	695 (35.1)	412 (31.7)	283 (41.6)	<.001
Age at first exposure to football, mean (SD), y	11.77 (3.1)	11.91 (3.0)	11.52 (3.2)	.007
Missing data, No.	28	20	8	
Debut year (year of first professional play), mean	1980	1978	1983	.20
Missing data, No.	4	1	3	
No. of professional seasons, mean (SD)	6.6 (3.9)	6.5 (3.9)	6.7 (3.9)	.26
Use of performance-enhancing drugs	308 (15.6)	174 (13.4)	134 (19.7)	<.001
Concussion signs and symptoms score, mean (SD)	28.7 (25.7)	22.55 (21.7)	40.3 (28.5)	<.001
Missing data, No.	36	28	8	
Former lineman	689 (34.8)	437 (33.6)	252 (37.0)	.14
Told they had CTE by a physician or medical professional	92 (4.6)	7 (0.5)	85 (12.5)	<.001
Depressive symptoms without suicidality (PHQ-8 score), mean (SD)	4.7 (5.4)	3.1 (4.0)	7.7 (6.2)	<.001
Depressive symptoms (PHQ-9 total score), mean (SD)	4.87 (5.7)	3.15 (4.1)	8.15 (6.7)	<.001
Suicidality (binary per PHQ-9 item 9)	235 (12.0)	64 (5.0)	171 (25.4)	<.001
Missing data, No.	24	17	7	
PHQ-9 item 9: thoughts of self-harm or hurting oneself				
Not at all	1721 (88.0)	1218 (95.0)	503 (74.6)	<.001
Several days	169 (8.6)	53 (4.1)	116 (17.2)	
More than half the days	38 (1.9)	6 (0.5)	32 (4.7)	
Nearly every day	28 (1.4)	5 (0.4)	23 (3.4)	
Missing data, No.	24	17	7	
Perceived cognitive function, mean (SD)	42.7 (9.6)	45.8 (8.5)	36.8 (8.8)	<.001
Emotional and behavioral dyscontrol, mean (SD)	48.2 (11.1)	45.0 (9.8)	54.4 (11.0)	<.001
Missing data, No.	17	10	7	
Anxiety symptoms (GAD-7 score), mean (SD)	3.6 (4.8)	2.3 (3.6)	6.2 (5.6)	<.001
Pain intensity score, mean (SD)	4.1 (2.1)	3.7 (1.9)	4.9 (2.1)	<.001
Missing data, No.	118	92	26	
Takes prescription pain medication	363 (18.4)	200 (15.4)	163 (23.9)	<.001
Missing data, No.	2	2	0	
Medication ever recommended for the following				
Headaches	185 (9.3)	59 (4.5)	126 (18.5)	<.001
ADHD	140 (7.1)	58 (4.5)	82 (12.0)	<.001
Diabetes	211 (10.7)	128 (9.9)	83 (12.2)	.11
High cholesterol	657 (33.2)	432 (33.3)	225 (33.0)	.92
Hypertension	719 (36.3)	485 (37.3)	234 (34.4)	.19
Low testosterone level	311 (15.7)	151 (11.6)	160 (23.5)	<.001
Reported the following diagnosis				
Vascular dementia	37 (1.9)	9 (0.7)	28 (4.1)	<.001
Alzheimer disease	31 (1.6)	8 (0.6)	23 (3.4)	<.001
Another type of dementia	108 (5.5)	26 (2.0)	82 (12.0)	<.001
Sleep apnea	607 (30.7)	349 (26.9)	258 (37.9)	<.001

Abbreviations: ADHD, attention-deficit/hyperactivity disorder; CTE, chronic traumatic encephalopathy; GAD-7, 7-item Generalized Anxiety Disorder questionnaire; PHQ-8, PHQ-9, 8-item or 9-item Patient Health Questionnaire.

^a^ Participants chose the category that best described their race and ethnicity, which were then categorized into the groups shown in the table.

^b^ Calculated as weight in kilograms divided by height in meters squared.

**Figure 1.**
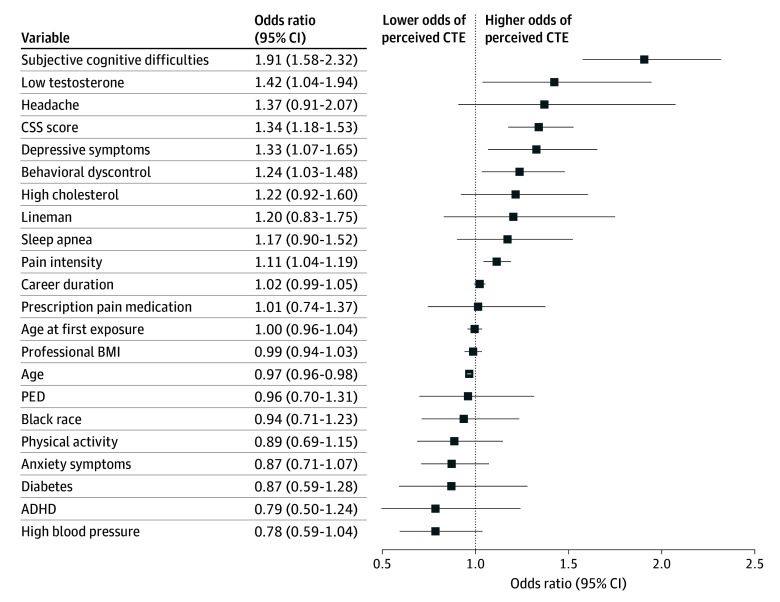
Demographic Variables, Football-Related Exposures, and Current Health Factors Associated With Perceived Chronic Traumatic Encephalopathy (CTE) Odds ratios and 95% CIs from the model of the association between demographic factors, football exposures, and health-related factors and perceived CTE. Note that data for those who were assigned the race and ethnicity category Native Hawaiian/Pacific Islander/Asian/American Indian/Alaskan Native or missing were removed from the image but not the model. Concussion signs and symptoms (CSS) score, anxiety symptoms, depressive symptoms, body mass index (BMI), emotional and behavioral dyscontrol, and perceived cognitive function are shown in standard deviation units. ADHD indicates attention-deficit/hyperactivity disorder; PED, performance-enhancing drugs.

Fully adjusted multivariable logistic regression identified demographic, football-related, and current health factors that were associated with perceived CTE (Figure 1). Subjective cognitive difficulties were the strongest predictor of perceived CTE (OR per 1 SD unit, 1.91; 95% CI, 1.58-2.31; *P* < .001), followed by low testosterone level (OR, 1.42; 95% CI, 1.04-1.94; *P* = .03), CSS score (OR per SD unit, 1.34; 95% CI, 1.18-1.53; *P* < .001), depressive symptoms (OR per 1 SD unit, 1.33; 95% CI, 1.07-1.65; *P* = .01), emotional and behavioral dyscontrol symptoms (OR per 1 SD unit, 1.24; 95% CI, 1.03-1.48; *P* = .02), and pain intensity (OR, 1.11; 95% CI, 1.04-1.19; *P* = .001). Older age (OR per SD unit, 0.97; 95% CI, 0.96-0.98; *P* < .001) was significantly associated with lower odds of perceived CTE. When loss of consciousness was used as a single measure of head injury exposure in multivariable analyses, results were similar (eFigure 1 in Supplement 1). Secondary analyses that included self-reported dementia-related diagnoses were essentially unchanged (eFigures 2 and 3 in Supplement 1).

**Figure 2.**
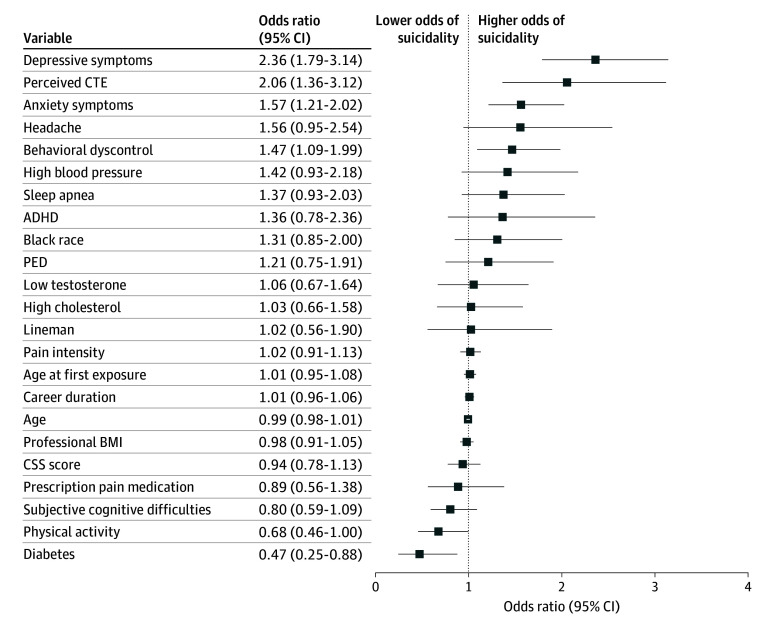
Demographic Factors, Football-Related Exposures, and Current Health Factors Associated With Suicidality Odds ratios and 95% CIs from the model of the association between demographic factors, football exposures, and health-related factors and perceived chronic traumatic encephalopathy (CTE). Note that data for those who were assigned the race and ethnicity category Native Hawaiian/Pacific Islander/Asian/American Indian/Alaskan Native or missing were removed from the image but not the model. Concussion signs and symptoms (CSS) score, anxiety symptoms, depressive symptoms, body mass index (BMI), emotional and behavioral dyscontrol, and perceived cognitive function are shown in standard deviation units. ADHD indicates attention-deficit/hyperactivity disorder; PED, performance-enhancing drugs.

Suicidality was reported by 171 of 681 participants with perceived CTE (25.4%) and 64 of 1299 without perceived CTE (5.0%). In multivariate models, depressive symptoms measured by the PHQ-8 (OR per SD unit, 2.36; 95% CI, 1.79-3.14; *P* < .001), perceived CTE (OR, 2.1; 95% CI, 1.21-2.02; *P* < .001), anxiety symptoms (OR per SD unit, 1.57; 95% CI, 1.21-2.02; *P* < .001), and emotional and behavioral dyscontrol (OR per SD unit, 1.47; 95% CI, 1.09-1.99; *P* = .01) were independently associated with suicidality (Figure 2). Meeting physical activity recommendations (OR, 0.68; 95% CI, 0.46-1.0; *P* = .05) and having diabetes (OR, 0.47; 95% CI, 0.25-0.88; *P* = .02) were associated with reduced odds of suicidality. After adjustment for established predictors of suicidality, participants with perceived CTE were twice as likely to report suicidal or self-harm thoughts (OR, 2.06; 95% CI, 1.36-3.12; *P* < .001). In multinomial logistic regression models that used multiple frequency categories for suicidality as the dependent variable, participants with perceived CTE and higher depressive symptom scores were more likely to report more frequent suicidal thoughts (eTable 2 in Supplement 1). Additional analysis included the dementia-related diagnoses and calculated similar results with suicidality (eFigure 3 in Supplement 1). Applying inverse probability weights to account for dropout prior to follow-up yielded comparable results with unweighted models (eTable 3 in Supplement 1).

**Figure 3.**
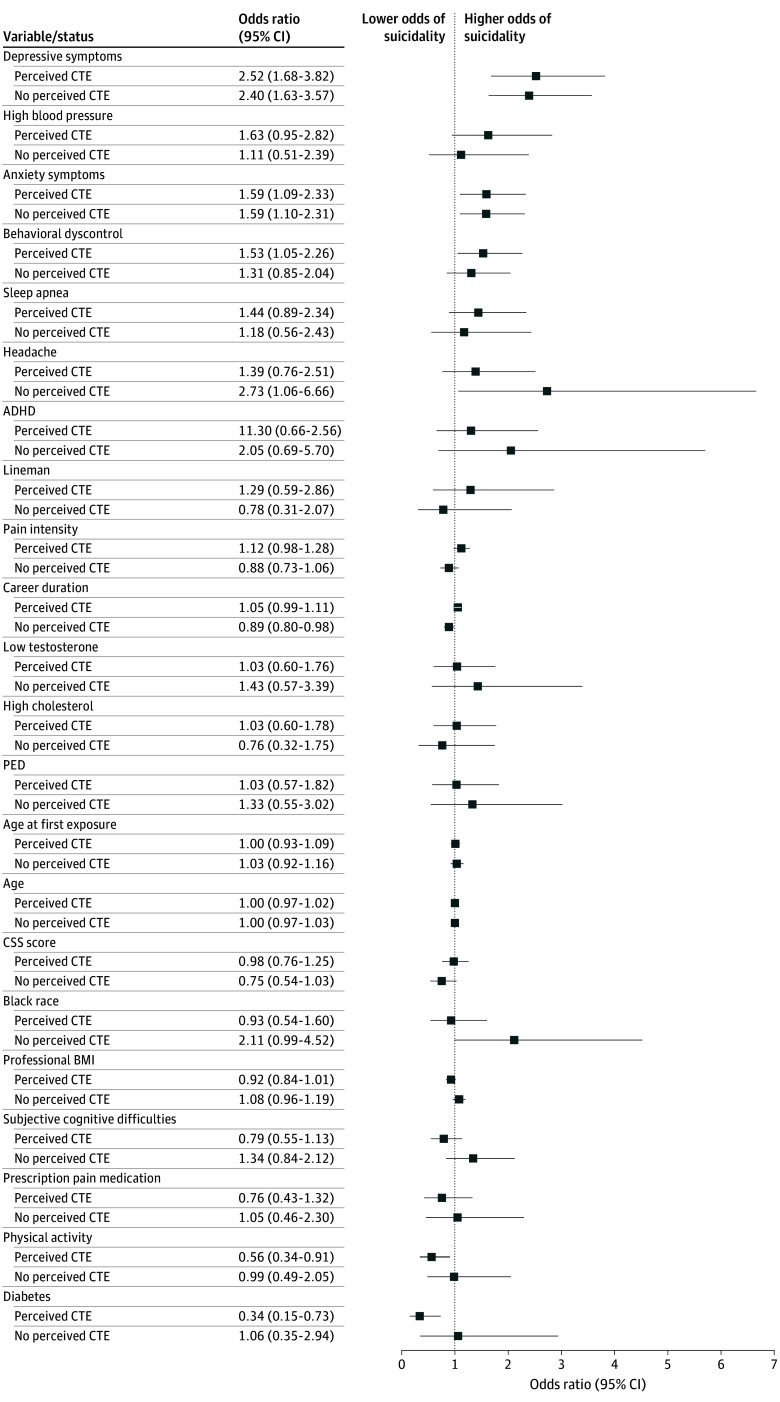
Demographic Factors, Football-Related Exposures, and Current Health Factors Associated With Suicidality and Stratified by Perceived Chronic Traumatic Encephalopathy (CTE) Odds ratios and 95% CIs from the model of the association between demographic factors, football exposures, and health-related factors and perceived CTE. Note that data for those who were assigned the race and ethnicity category Native Hawaiian/Pacific Islander/Asian/American Indian/Alaskan Native or missing were removed from the image but not the model. Concussion signs and symptoms (CSS) score, anxiety symptoms, depressive symptoms, body mass index (BMI), emotional and behavioral dyscontrol, and perceived cognitive function are shown in standard deviation units. ADHD indicates attention-deficit/hyperactivity disorder; PED, performance-enhancing drugs.

To determine whether factors that predict suicidality differed among men with and without perceived CTE, we performed multivariable logistic regression models to predict suicidality stratified by perceived CTE status (Figure 3). Among men with perceived CTE, depression (OR per SD unit, 2.52; 95% CI, 1.68-3.82, *P* < .001), anxiety (OR per SD unit, 1.59; 95% CI, 1.09-2.33; *P* = .02), and emotional and behavioral dyscontrol (OR per SD unit, 1.53; 95% CI, 1.05-2.26; *P* = .03) continued to significantly predict suicidality, while diabetes (OR, 0.34; 95% CI, 0.15-0.73; *P* = .01) and physical activity (OR, 0.56, 95% CI, 0.34-0.91, *P* = .02) remained associated with lower odds of suicidality. In contrast, among men without perceived CTE, headaches were the most significant predictor of suicidality (OR, 3.00; 95% CI, 1.17-7.38; *P* = .02), followed by depressive symptoms (OR per SD unit, 2.40; 95% CI, 1.64-3.5; *P* < .001) and anxiety (OR per SD, 1.49; 95% CI, 1.05-2.11; *P* = .03). Longer careers were associated with lower odds of endorsing suicidal ideation among men without perceived CTE (OR, 0.89; 95% CI, 0.80-0.98; *P* = .03). Wide confidence intervals in those without perceived CTE were likely attributable to the relatively small number of suicidality cases in the subgroup without perceived CTE (n = 64/1282; 5.0%).

## Discussion

This study examined the proportion of and clinical correlates of perceived CTE among living former professional ASF players. Approximately one-third of former players (34.4%) reported perceived CTE. Perceived CTE was associated with younger age, CSS scores, and postcareer problems, including depression, subjective cognitive difficulties, emotional and behavioral dyscontrol, pain, and low testosterone level. Importantly, among former players with perceived CTE, the proportion reporting suicidality was 5-fold greater than in those without perceived CTE. In aggregate, these data establish a high rate of perceived CTE among former professional ASF players and illustrate that this perception is associated with mental health problems, including suicidality.

To date, CTE-NC identified on autopsy has not been definitively linked to proposed antemortem depression, anxiety, or suicidality.^4,6^ Nonetheless, mainstream media pieces and published articles related to individual players routinely implicate “CTE” as the underlying cause of suicide and psychiatric problems among former professional ASF players.^7,8,9,10,11,12,13^ While the exact effect of this media coverage remains uncertain, it is possible that this has contributed to former ASF players and their physicians^37^ concluding that CTE is directly responsible for myriad postcareer health problems. Taken together, it is critical that the results presented here not contribute to personal, scientific, or societal misattribution of suicidal ideation as a premortem manifestation of CTE-NC. Our findings suggest that some suicidality may stem from the assumption that a former player has an untreatable neurodegenerative disease. These findings further affirm the need to identify both clinical correlates and preventive measures for CTE-NC while also underscoring the potential for misattribution of symptoms that can have devastating consequences for players and their families.

Definitive conclusions regarding why former ASF players in this cohort report perceived CTE cannot be made. However, several plausible synergistic possibilities deserve consideration. First, clinical characteristics associated with perceived CTE among former professional ASF players may truly reflect antemortem manifestations of CTE-NC. However, it is noteworthy that depression and anxiety, 2 clinical conditions strongly associated with perceived CTE in this study, have previously been shown not to represent clinical manifestations of CTE-NC.^4,6^ Alternatively, perceived CTE may arise because of the presence of symptoms caused by alternative pathological processes, which may be related to a history of repetitive head impacts.^46,47^ As shown in the Table, nearly 20% of players with perceived CTE reported being diagnosed with Alzheimer disease, vascular dementia, or another dementia compared with about 3% of the group without perceived CTE. It is possible that the clinical findings and symptoms that led a health care professional to diagnose dementia may be caused, at least in part, by CTE-NC. Conversely, former professional football players may have a different form of dementia but perceive themselves as having CTE-NC. These issues underscore the clinical imperative to exclude all alternative forms of neurologic, psychiatric, or medical conditions (especially those that are treatable or reversible) among people at risk for CTE-NC. Finally, living former professional ASF players may have perceived CTE in the absence of clinical symptoms. Repeated exposure to the highly publicized link between ASF participation and subsequent brain disease may lead otherwise healthy former players to conclude that CTE-NC is an inevitable consequence of prior ASF participation.^48^ Future work will be required to reconcile the relative contributions of these hypotheses.

Suicide is a devastating public health crisis. This study suggests that 1 in 8 former professional ASF players (12.0%) experience suicidality, which was largely accounted for by men with perceived CTE. After adjustment for well-established suicidality risk factors, including symptoms of depression, anxiety, and emotional dyscontrol, perceived CTE remained an independent determinant of increased risk of suicidality in former players. Accordingly, the identification of men with perceived CTE may represent an opportunity to enhance the value and accuracy of suicide risk screening in this population. Such surveillance may be particularly salient in Black former players, given recent increasing trends in suicide among Black men in the United States.^49^ Our data provide novel insights into clinical factors, including perceived CTE, that are associated with suicidality and may inform risk stratification, diagnostic assessment, and therapeutic intervention in former professional ASF players. Certain modifiable lifestyle behaviors may be protective against suicidality and perceived CTE, as seen in the association of reduced risk with increased physical activity. Additional research into associations with exercise and the counterintuitive reduced odds of suicidality seen with diabetes would be of interest to those providing medical care for at-risk individuals.

### Limitations

This study has several limitations. First, we emphasize that our data cannot be used to determine the degree to which true underlying CTE-NC explains the clinical profile of men reporting perceived CTE or whether perceived CTE stems from misattribution of symptoms caused by alternative disease processes. This represents an area of critically important future work that will only be possible when accurate tools to diagnose premortem CTE-NC have been developed. Second, the comorbidities that were associated with perceived CTE were self-reported. However, many of these comorbidities, including anxiety, depression, and emotional and behavioral dyscontrol, are self-reported by nature, and we used clinically validated measures to establish their presence. Third, we acknowledge the possibility of a biased sample and that our cohort may not be fully representative of all former professional ASF players. However, being more or less likely to join our study because of perceiving CTE would not account for the observed association between perceived CTE and suicidality. For that, there would have had to be preferential participation in our study by people with perceived CTE and suicidality or without both. Further arguing against this is that the same potential bias could occur from selective dropout between the baseline and follow-up surveys where perceived CTE and suicidality were assessed. However, we addressed this possibility through inverse probability weighting for dropout and found no evidence of selection bias. Fourth, 1 in 5 of those with perceived CTE reported being diagnosed with either Alzheimer disease, vascular dementia, or another dementia; we do not have independent verification of those diagnoses, and their accuracy cannot be determined. Finally, data on suicidality for this study did not distinguish the extent of suicidality (eg, ideation, intent, imminence), and we therefore cannot exclude the possibility of misclassification of suicidality beyond what has been reported here.

## Conclusions

In this study, one-third of former professional ASF players reported perceived CTE. Data from this study establish differential clinical correlates for men with and without perceived CTE, thereby offering insights into the basis of this common perception. Future work will be required to determine the degree to which perceived CTE aligns with the true presence of this pathology as substantiated by neuropathological evidence or a future antemortem test. In the interim, our data suggest perceived CTE may represent a marker of increased risk of suicidality. Consideration of this easily ascertainable factor may direct diagnostic and therapeutic interventions and may enhance the detection of suicide risk among former professional ASF players.

## Notes

Supplement 1.
eMethods
**eTable 1.** Demographic factors, football-related exposures, and health-related characteristics of responders included in the analyses compared to all FPHS participants **eTable 2.** Adjusted odds ratios, 95% confidence intervals, and p-values for multinomial models predicting the frequency of suicidality **eTable 3.** Adjusted odds ratios, 95% confidence intervals and p-values from models of suicidality using inverse probability weighting (n=1,978) **eFigure 1.** Demographic factors, football-related exposures, and current health factors associated with perceived CTE in models that use number of loss of consciousness episodes (LOC) instead of concussion signs and symptom scores **eFigure 2.** Demographic factors, football-related exposures, and current health factors associated with perceived CTE including dementia-related diagnoses **eFigure 3.** Demographic factors, football-related exposures, and current health factors associated with suicidality including dementia-related diagnoses
eReferences

Supplement 2. Data sharing statement
