# Supplementary material for: Perceived Chronic Traumatic Encephalopathy and Suicidality in Former Professional Football Players
Source: JAMA Neurol. 2024 Sep 23;81(11):1130–9. doi: 10.1001/jamaneurol.2024.3083 (PMC11420824; doi:10.1001/jamaneurol.2024.3083)
Supplement: Supplement 1. — eMethods eTable 1. Demographic factors, football-related exposures, and health-related characteristics of responders included in the analyses compared to all FPHS participants eTable 2. Adjusted odds ratios, 95% confidence intervals, and p-values for multinomial models predicting the frequency of suicidality eTable 3. Adjusted odds ratios, 95% confidence intervals and p-values from models of suicidality using inverse probability weighting (n=1,978) eFigure 1. Demographic factors, football-related exposures, and current health factors associated with perceived CTE in models that use number of loss of consciousness episodes (LOC) instead of concussion signs and symptom scores eFigure 2. Demographic factors, football-related exposures, and current health factors associated with perceived CTE including dementia-related diagnoses eFigure 3. Demographic factors, football-related exposures, and current health factors associated with suicidality including dementia-related diagnoses eReferences [file jamaneurol-e243083-s001.pdf]

Supplemental Online Content

Grashow R, Terry DP, Iverson GL, et al. Perceived chronic traumatic encephalopathy and suicidality in former professional football players. *JAMA Neurol*. Published online September 23, 2024. doi:10.1001/jamaneurol.2024.3083

eMethods

eTable 1. Demographic factors, football-related exposures, and health-related characteristics of responders included in the analyses compared to all FPHS participants

eTable 2. Adjusted odds ratios, 95% confidence intervals, and p-values for multinomial models predicting the frequency of suicidality

eTable 3. Adjusted odds ratios, 95% confidence intervals and p-values from models of suicidality using inverse probability weighting (n=1,978)

eFigure 1. Demographic factors, football-related exposures, and current health factors associated with perceived CTE in models that use number of loss of consciousness episodes (LOC) instead of concussion signs and symptom scores

eFigure 2. Demographic factors, football-related exposures, and current health factors associated with perceived CTE including dementia-related diagnoses

eFigure 3. Demographic factors, football-related exposures, and current health factors associated with suicidality including dementia-related diagnoses

eReferences

This supplemental material has been provided by the authors to give readers additional information about their work.

© 2024 Grashow R et al. *JAMA Neurology.*

eMethods

Retrospective head injury measures

To assess concussion burden, participants were asked, "While playing or practicing football, did you experience a blow to the head, neck, or upper body followed by any of the following symptoms: headaches, nausea, dizziness, loss of consciousness, memory problems, disorientation, confusion, seizure, visual problems, or feeling unsteady of your feet?" For each of these signs/symptoms, participants chose "none," "once," "2 to 5 times," "6 to 10 times," or "11 times". These summed to create CSS scores as previously described.[1-4] Total number of loss of consciousness episodes were considered a stand-alone proxy of number of sport-related traumatic brain injuries in sensitivity analyses.[5-9]

Comorbidity ascertainment

Sleep apnea, stroke, CTE, Alzheimer's dementia, vascular dementia, and other dementia were based on a yes/no response to the question: "Has a health care provider ever told you that you have had any of the following diagnoses or health outcomes?" Participants were asked "Has a medical provider ever recommended or prescribed medication for [condition]" and "Are you currently taking medication [for that condition]?" for the following conditions: ADHD, diabetes, headaches, hypertension, hyperlipidemia, memory loss, and low testosterone.

Symptoms of Depression and Anxiety

Depression severity was assessed using the Patient Health Questionnaire (PHQ-9),[10] which asks participants to report whether they experienced the following nine symptoms over the prior two weeks: 1) hopelessness; 2) reduced pleasure in activities; 3) trouble sleeping or falling asleep; 4) low energy; 5) changes in appetite; 6) feelings of failure; 7) trouble concentrating; 8) feelings of lethargy or restlessness; and 9) having thoughts that one would be better off dead or of harming oneself. For each symptom, participants were given the following options: a) not at all; b) several days; c) more than half the days; d) nearly every day, which were assigned the numerical values of 0, 1, 2, 3 respectively. For analyses that explored associations with perceptions of CTE, scores from all nine questions were summed to calculate depression severity. In analyses that explored suicidality as the dependent variable, suicidality was assessed using the final PHQ-9 question that queries suicidality and self-harm was coded dichotomously, such that those who answered not at all or 0 were assigned as "no suicidality", with anyone reporting more than several days over the prior two weeks with thoughts of suicide or self-harm as "suicidality." In these analyses depression severity was assessed using the PHQ-8[11] to assess depressive symptoms from PHQ items that do not relate to suicide or self-harm. If participants were missing more than three PHQ questions, they were excluded. If they were missing one, two, or three responses, those values were imputed to be zero or "not at all." Imputation was done for 80 players missing one response, 11 players missing two responses, and two participant missing three responses.

Anxiety severity was assessed using the Generalized Anxiety Disorder (GAD-7) questionnaire[12]. The GAD-7 queries seven anxiety symptoms experienced over the previous two weeks including 1) feeling nervous or anxious; 2) unable to stop worrying; 3) worrying too much about different things; 4) trouble relaxing; 5) feelings of restlessness; 6) irritability; and 7) concern that something awful might happen. As described above for the PHQ-9, participants selected from the following options: a) not at all; b) several days; c) more than half the days; d) nearly every day, also assigned the numerical values of 0, 1, 2, 3 respectively. If participants were missing more than three GAD questions, they were excluded. If they were missing one, two, or three responses, those values were imputed to be zero or "not at all". Imputation was done for 42 players missing one response, two players missing two responses, and one participant missing three responses.

© 2024 Grashow R et al. *JAMA Neurology.*

Inverse probability weighting

We implemented inverse probability weighting for dropout from the baseline survey to the follow-up to assess potential selection bias. Specifically, we predicted participation in the second survey using demographic, football-related, and current health variables. We inverted each of the predicted probabilities and calculated stabilized weights[13] by replacing the numerator with the probability of falling into that completion group. The median (interquartile range) of weights for the follow-up group was 0.96 (0.84, 1.20), and 1.01 (0.81, 1.28) for those without follow-up. We excluded two participants with follow-up survey who had stabilized weights outside the range of weights among those not in the follow-up survey (less than 0.51 or greater than 18.64), and reran the fully-adjusted model of suicidality using the weights for dropout.

© 2024 Grashow R et al. *JAMA Neurology.*

eTable 1: Demographic factors, football-related exposures, and health-related characteristics of responders included in the analyses compared to all FPHS participants.

		Follow up survey status	
	**Total (N=4,189)**	**Baseline only (N=2,209)**	**Baseline and follow-up (N=1,980)**
Demographics			
**Age at baseline, Mean (SD)**	51. 8 (14.4)	50.8 (15.0)	52.9 (13.7)
**Race**			
Black	1,634 (39.0%)	1,039 (47.0%)	595 (30.1%)
Missing	53 (1.3%)	29 (1.3%)	24 (1.2%)
Other	126 (3.0%)	68 (3.1%)	58 (2.9%)
White	2,376 (56.7%)	1,073 (48.6%)	1,303 (65.8%)
**Body mass index, Mean (SD)**	31.3 (5.0)	31.6 (5.2)	31.0 (4.8)
N-Miss	30	20	10
Football-related factors			
**Linemen status**	1,420 (33.9%)	731 (33.1%)	689 (34.8%)
**Number of seasons, Mean (SD)**	6.7 (3.9)	6.7 (3.8)	6.6 (3.9)
**Concussion signs and symptoms score, Mean (SD)**	30.7 (27.2)	32.6 (28.3)	28.7 (25.7)
N-Miss	91	55	36
**Use of performance enhancing drugs during career**	656 (15.7%)	348 (15.8%)	308 (15.6%)
Health-related factors			
**Low testosterone**	730 (17.9%)	416 (19.3%)	314 (16.3%)
N-Miss	102	52	50
**Takes Rx pain medication**	1,151 (27.5%)	627 (28.4%)	524 (26.5%)
**Perceived cognitive function, Mean (SD)**	40.6 (9.8)	39.3 (9.8)	42.0 (9.6)
N-Miss	10	5	5
**Told they have CTE by a medical provider**	121 (2.9%)	78 (3.5%)	43 (2.2%)
**AD/Dementia**	147 (3.5%)	111 (5.0%)	36 (1.8%)
**Depression symptoms, Mean (SD)**	1.4 (1.7)	1.6 (1.8)	1.2 (1. 6)
N-Miss	8	6	2
**Anxiety symptoms, Mean (SD)**	1.6 (1.7)	1.8 (1.8)	1.3 (1.5)
N-Miss	8	6	2
**Sleep apnea**	940 (22.4%)	519 (23.5%)	421 (21.3%)

Note: CTE=chronic traumatic encephalopathy; N-Miss=number of subjects with missing data; SD=standard deviation; AD=Alzheimer's disease.

© 2024 Grashow R et al. *JAMA Neurology.*

eTable 2: Adjusted odds ratios, and 95% confidence intervals for multinomial models predicting the frequency of suicidality. Numbers of cases and controls for each categorical variable are shown, and N, mean and standard deviation (SD) are shown for continuous variables.

**Variable**	**Not at all N= 1721**	**Several days N = 169**	**More than half the days N = 38**	**Every day N = 28**
Perceived CTE				
Yes/No	503/1218	53/116	6/32	5/23
Odds ratio,95% CI	Reference	2.0, 1.3-3.0**	3.4, 1.2-9.8*	3.7, 0.6-24.3
Headache				
Yes/No	113/1608	53/116	12/26	7/21
Odds ratio,95% CI	Reference	1.8, 1.1-2.9*	0.9, 0.3-2.5	0.18, 0.0-1.1
Behavioral dyscontrol				
Mean (SD)	1721, 46.5 (10.1)	59.3 (8.9)	63.5 (8.6)	69.2 (11.7)
Odds ratio,95% CI	Reference	1.6, 1.2-2.2, **	1.1, 0.6-2.2	1.5, 0.5-4.4
Sleep apnea				
Yes/No	487/1234	84/85	15/23	14/14
Odds ratio,95% CI	Reference	1.6, 1.0-2.3*	0.6, 0.2-1.4	1.1, 0.2-4.8
Race: Other				
Yes/No	44/1169	7/88	2/15	2/15
Odds ratio,95% CI	Reference	1.5, 0.5-4.2	3.4, 0.5-23.0	12.1, 0.5-301.2
High blood pressure				
Yes/No	604/1117	76/93	18/20	12/16
Odds ratio,95% CI	Reference	1.3, 0.9-2.1	2.2, 0.9-5.7	2.3, 0.47-11.0
ADHD				
Yes/No	87/1634	32/137	13/25	7/21
Odds ratio,95% CI	Reference	1.3, 0.7-2.3	1.9, 0.7-5.2	0.8, 0.2-4.7
Race: Black				
Yes/No	486/1169	73/88	21/15	10/15
Odds ratio,95% CI	Reference	1.3, 0.8-2.0	2.8, 1.0-7.7*	0.7, 0.2-3.8
Performance enhancing drugs				
Yes/No	243/1478	38/131	10/28	11/17
Odds ratio,95% CI	Reference	1.2, 0.7-1.9	1.1, 0.4-3.1	4.2, 0.9-20.3
Depressive symptoms				
Mean (SD)	1721, 3.6 (4.1)	10.6 (5.1)	16.3 (4.6)	21.6 (3.3)
Odds ratio,95% CI	Reference	1.2, 1.1-1.2***	1.3, 1.1-1.5***	2.0, 1.5-2.6***
Lineman status				
Yes/No	593/1128	64/105	12/26	10/18
Odds ratio,95% CI	Reference	1.1, 0.6-2.1	0.4, 0.1-1.6	1.1, 0.1-11.0
Anxiety				
Mean (SD)	1721, 2.7 (3.7)	13.6 (4.4)	13.6, 4.4)	17.0 (5.2)
Odds ratio,95% CI	Reference	1.1, 1.0-1.2**	1.2, 1.0-1.3**	1.1, 0.9-1.4
High cholesterol				
Yes/No	569/1152	61/108	15/23	5/23
Odds ratio,95% CI	Reference	1.1, 0. 7-1. 7	1.2, 0.5-3.1	0.2, 0.0-1.5
Pain intensity				
Mean (SD)	1721, 3.9 (2.0)	5.4, (1.9)	6.2 (2.0)	5.7 (2.5)
Odds ratio,95% CI	Reference	1.0, 0.9-1.12	1.0, 0.8-1.3	0.8, 0.5-1.3

© 2024 Grashow R et al. *JAMA Neurology.*

Age of first football exposure				
Mean (SD)	1721, 11.8 (3.0)	11.6 (2.9)	11.9 (3.6)	10.8 (4.9)
Odds ratio,95% CI	Reference	1.0, 0.9-1.1	1.1, 1.0-1.3	1.0, 0.8-1.2
Career Duration				
Mean (SD)	1721, 6.6 (3.9)	6.5 (3.9)	6.8 (3.6)	6.5 (3.9)
Odds ratio,95% CI	Reference	1.0, 1.0-1.1	1.0, 0.9-1.2	1.0, 0.8-1.2
CSS				
Mean (SD)	1721, 26.5 (23.9)	43.4 (32.3)	47.9 (30.7)	55.3 (27.4)
Odds ratio,95% CI				
Age	Reference	1.0, 1.0-1.0	1.0, 1.0-1.0	1.0, 1.0-1.0
Mean (SD)	1721, 58.1 (13.9)	53.9 (12.7)	50.9 (12.4)	54.1 (13.1)
Odds ratio,95% CI	Reference	1.0 1.0-1.0	1.0, 0.9-1.0	1.0, 0.9-1.0
Low testosterone				
Yes/No	233/1488	47/122	16/22	8/20
Odds ratio,95% CI	Reference	1.0, 0.6-1.6	2.1, 0.8-5.0	0.4, 0. 1-2.6
Professional BMI				
Mean (SD)	1721, 30.3 (4.0)	30.9 (4.5)	31.0 (4.0)	31.7 (4.7)
Odds ratio,95% CI	Reference	1.0, 0.9-1.1	1.1, 0.9-1.3	1.0, 0.7-1.3
Rx pain medication				
Yes/No	288/1433	52/117	11/27	10/18
Odds ratio,95% CI	Reference	0.9, 0.6-1.4	0.7, 0.2-1.8	1.7, 0.3-8.4
Subjective cognitive difficulties				
Mean (SD)	1721, 44.0 (8.9)	34.9 (8.4)	29.7 (6.7)	25 (6.1)
Odds ratio,95% CI	Reference	0.8, 0.6-1.1	1.0, 0.5-2.0	1.1, 0.3-4.1
Physical activity				
Yes/No	1167/554	78/91	19/19	9/19
Odds ratio,95% CI	Reference	0.6, 0.4-0.9*	1.1, 0.4-2.6	0.6, 0.1-3.0
Diabetes				
Yes/No	182/1593	19/150	4/34	4/24
Odds ratio,95% CI	Reference	0.5, 0.2-0.9*	0.2, 0.1-0.9*	1.1, 0.1-9.6

Note: CTE=chronic traumatic encephalopathy; CI=confidence interval; CSS=Concussion signs and symptoms; BMI=body mass index; PED=performance enhancing drugs; ADHD=attention-deficit/hyperactivity disorder; Rx=prescription. *Indicates statistical significance p<0.05, ***indicates statistical significance, p<0.001

eTable 3: Adjusted odds ratios, 95% confidence intervals and p-values from models of suicidality using inverse probability weighting (n=1,978).

Term	Odds ratio	95% CI	P-value
Perceived CTE	2.18	1.56, 3.05	p<0.001
Depressive symptoms	2.16	1.73, 2.71	p<0.001
Anxiety symptoms	1.69	1.37, 2.08	p<0.001
Hypertension	1.65	1.16, 2.34	0.01
Headache	1.64	1.12, 2.4	0.01
ADHD	1.58	1.03, 2.41	0.04
Race: Other	1.57	0.68, 3.48	0.28
Emotional and behavioral dyscontrol	1.49	1.17, 1.9	0.001
Sleep apnea	1.47	1.06, 2.02	0.02
Race: Black	1.4	0.99, 1.97	0.06
PED	1.22	0.83, 1.77	0.31
High cholesterol	1.1	0.78, 1.56	0.59
Lineman status	1.09	0.67, 1.8	0.72
Low testosterone	1.02	0.71, 1.46	0.92
Age of first exposure	1.02	0.97, 1.07	0.54
Career duration	1	0.96, 1.04	0.99
Age	1	0.99, 1.01	0.94
Pain intensity	0.98	0.9, 1.07	0.65
Professional BMI	0.98	0.92, 1.04	0.48
CSS	0.93	0.81, 1.08	0.35
Rx pain medication	0.83	0.57, 1.19	0.31
Subjective cognitive difficulties	0.79	0.62, 1.01	0.06
Physical activity	0.62	0.45, 0.84	0.002
Diabetes	0.39	0.23, 0.64	p<0.001
Race: Missing	0.32	0.02, 3.56	0.45

Note: CTE=chronic traumatic encephalopathy; CI=confidence interval; CSS=Concussion signs and symptoms; BMI=body mass index; PED=performance enhancing drugs; ADHD=attention-deficit/hyperactivity disorder; Rx=prescription.

© 2024 Grashow R et al. *JAMA Neurology.*

eFigure 1: Demographic factors, football-related exposures, and current health factors associated with perceived CTE in models that use number of loss of consciousness episodes (LOC) instead of concussion signs and symptom scores.

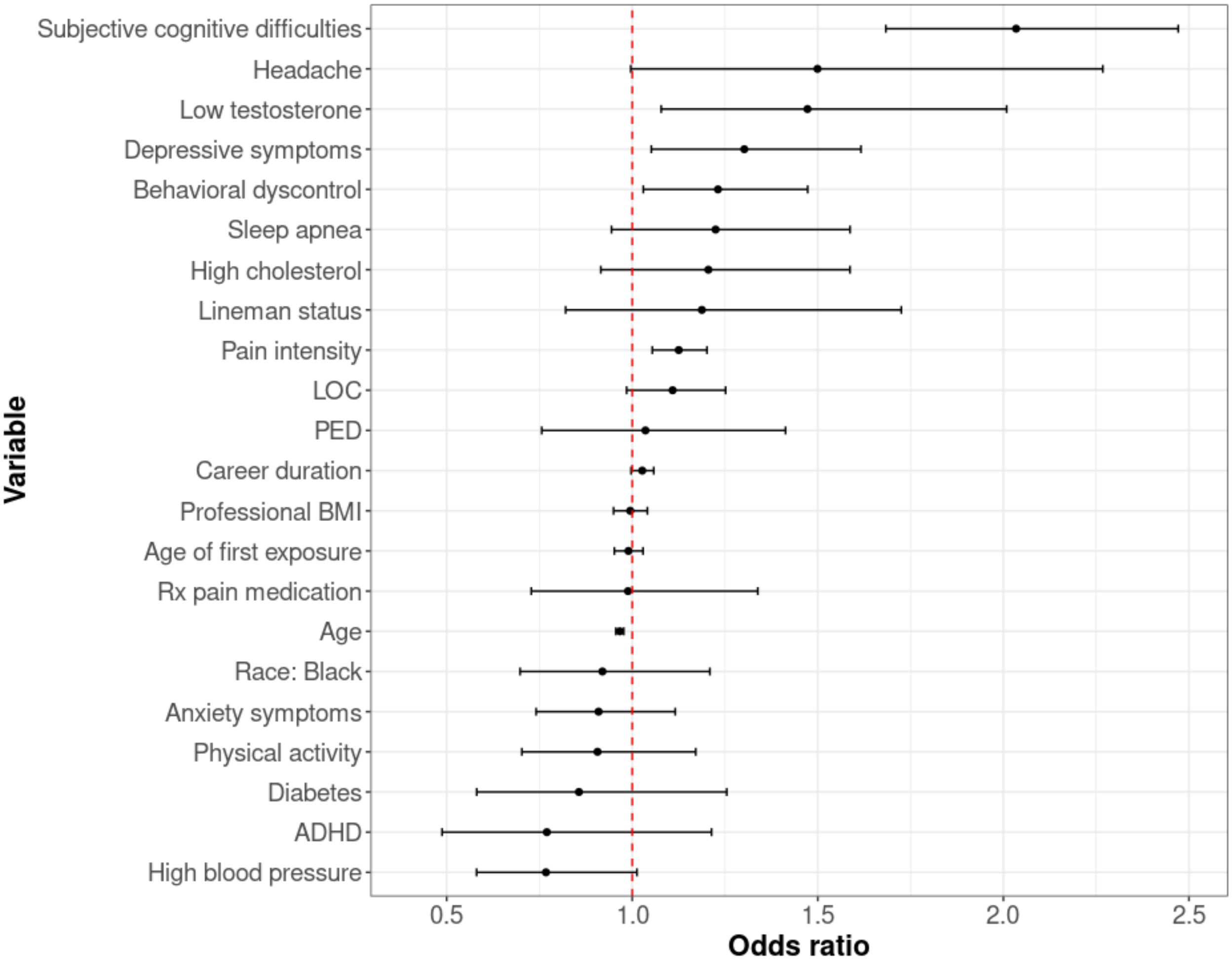

Figure legend: Odds ratios and 95% confidence intervals from the model of the association between demographic factors, football exposures, and health-related factors and perceived CTE. Note that those who assigned race categories including Native Hawaiian/Pacific Islander/Asian/American Indian/Alaskan Native or Missing have been removed from the image but not the model. Concussion signs and symptoms score, anxiety symptoms, depressive symptoms, body mass index (BMI), emotional and behavioral dyscontrol, and perceived cognitive function are shown in standard deviation units. CSS=Concussion signs and symptoms; CTE=chronic traumatic encephalopathy; BMI=body mass index; PED=performance enhancing drugs; ADHD=attention-deficit/hyperactivity disorder; Rx=prescription.

© 2024 Grashow R et al. *JAMA Neurology.*

eFigure 2. Demographic factors, football-related exposures, and current health factors associated with perceived CTE including dementia-related diagnoses

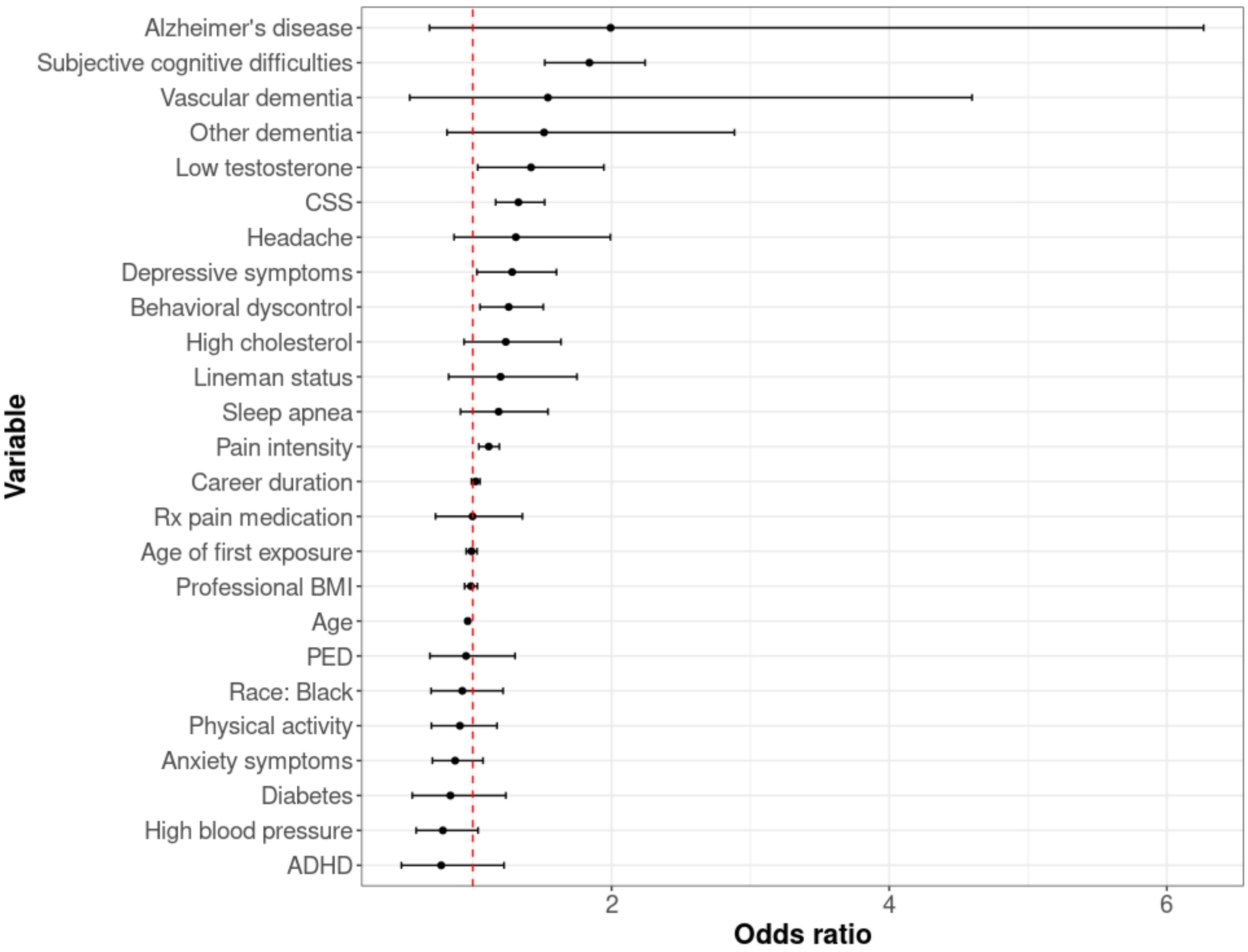

Figure legend: Odds ratios and 95% confidence intervals from the model of the association between demographic factors, football exposures, and health-related factors and perceived CTE. Note that those who assigned race categories including Native Hawaiian/Pacific Islander/Asian/American Indian/Alaskan Native or Missing have been removed from the image but not the model. Concussion signs and symptoms score, anxiety symptoms, depressive symptoms, body mass index (BMI), emotional and behavioral dyscontrol, and perceived cognitive function are shown in standard deviation units. CSS=Concussion signs and symptoms; CTE=chronic traumatic encephalopathy; BMI=body mass index; PED=performance enhancing drugs; ADHD=attention-deficit/hyperactivity disorder; Rx=prescription.

© 2024 Grashow R et al. *JAMA Neurology.*

eFigure 3. Demographic factors, football-related exposures, and current health factors associated with suicidality including dementia-related diagnoses.

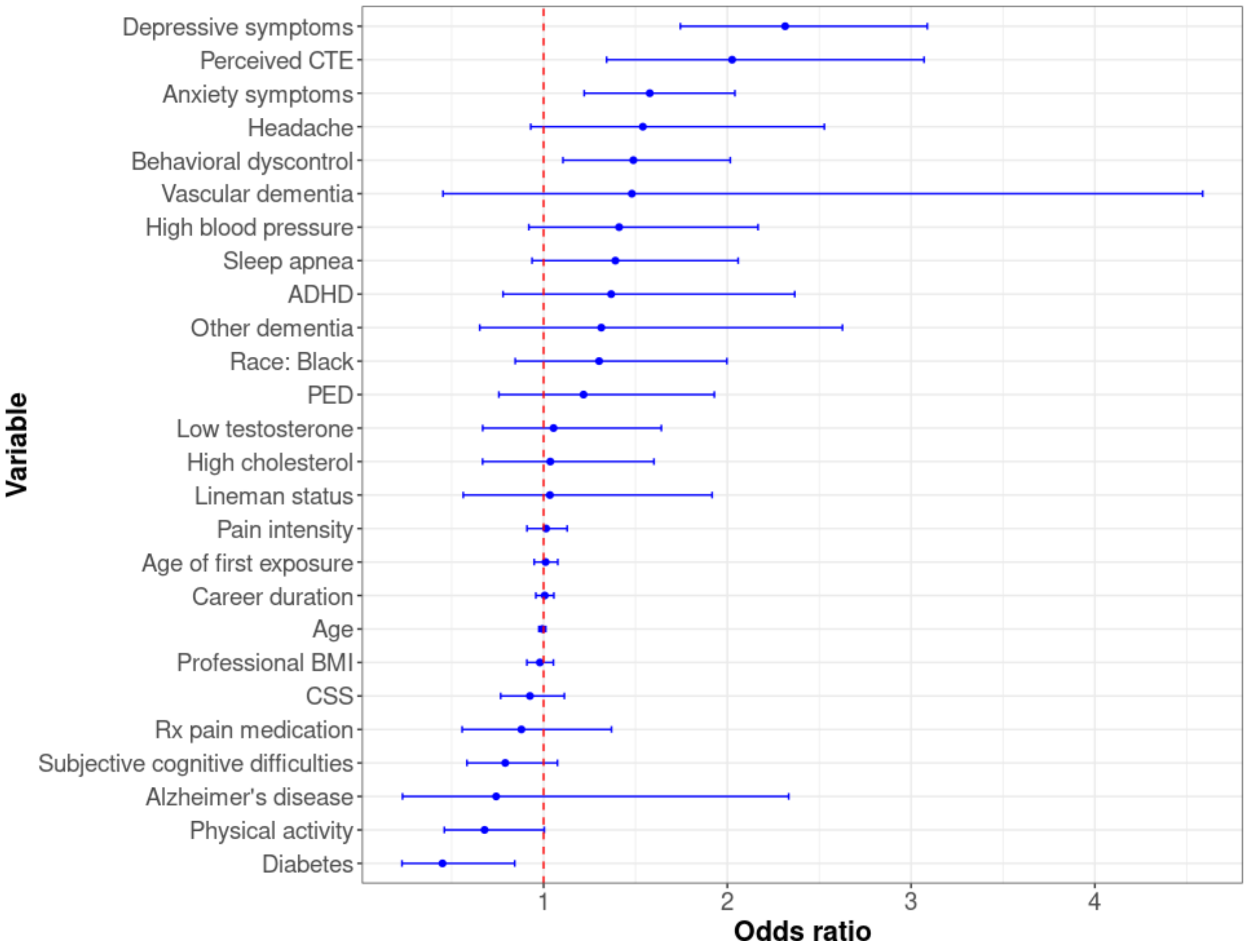

Figure legend: Odds ratios and 95% confidence intervals from the model of the association between demographic factors, football exposures, and health-related factors and perceived CTE. Note that those who assigned race categories including Native Hawaiian/Pacific Islander/Asian/American Indian/Alaskan Native or Missing have been removed from the image but not the model. Concussion signs and symptoms score, anxiety symptoms, depressive symptoms, body mass index (BMI), emotional and behavioral dyscontrol, and perceived cognitive function are shown in standard deviation units. CSS=Concussion signs and symptoms; CTE=chronic traumatic encephalopathy; BMI=body mass index; PED=performance enhancing drugs; ADHD=attention-deficit/hyperactivity disorder; Rx=prescription.

© 2024 Grashow R et al. *JAMA Neurology.*

eReferences

1. Grashow R, Weisskopf MG, Miller KK, et al. Association of Concussion Symptoms With Testosterone Levels and Erectile Dysfunction in Former Professional US-Style Football Players. *JAMA Neurol.* 2019;76(12):1428-1438.
2. Roberts AL, Pascual-Leone A, Speizer FE, et al. Exposure to American Football and Neuropsychiatric Health in Former National Football League Players: Findings From the Football Players Health Study. *Am J Sports Med.* 2019;47(12):2871-2880.
3. Roberts AL, Zafonte RD, Speizer FE, et al. Modifiable Risk Factors for Poor Cognitive Function in Former American-Style Football Players: Findings from the Harvard Football Players Health Study. *J Neurotrauma.* 2020.
4. Grashow R, Tan CO, Izzy S, et al. Association Between Concussion Burden During Professional American-Style Football and Postcareer Hypertension. *Circulation.* 2023;147(14):1112-1114.
5. Jackson GL, Hamilton NS, Tupler LA. Detecting traumatic brain injury among veterans of Operations Enduring and Iraqi Freedom. *N C Med J.* 2008;69(1):43-47.
6. Amen DG, Newberg A, Thatcher R, et al. Impact of playing American professional football on long-term brain function. *J Neuropsychiatry Clin Neurosci.* 2011;23(1):98-106.
7. Blyth BJ, Bazarian JJ. Traumatic alterations in consciousness: traumatic brain injury. *Emerg Med Clin North Am.* 2010;28(3):571-594.
8. Corrigan JD, Bogner J. Initial reliability and validity of the Ohio State University TBI Identification Method. *J Head Trauma Rehabil.* 2007;22(6):318-329.
9. Ramos SDS, Liddement J, Addicott C, Fortescue D, Oddy M. The development of the Brain Injury Screening Index (BISI): A self-report measure. *Neuropsychol Rehabil.* 2020;30(5):948-960.
10. Bauer AM, Chan YF, Huang H, Vannoy S, Unutzer J. Characteristics, management, and depression outcomes of primary care patients who endorse thoughts of death or suicide on the PHQ-9. *J Gen Intern Med.* 2013;28(3):363-369.
11. Wu Y, Levis B, Riehm KE, et al. Equivalency of the diagnostic accuracy of the PHQ-8 and PHQ-9: a systematic review and individual participant data meta-analysis - ERRATUM. *Psychol Med.* 2020;50(16):2816.
12. Spitzer RL, Kroenke K, Williams JB, Lowe B. A brief measure for assessing generalized anxiety disorder: the GAD-7. *Arch Intern Med.* 2006;166(10):1092-1097.
13. Chesnaye NC, Stel VS, Tripepi G, et al. An introduction to inverse probability of treatment weighting in observational research. *Clin Kidney J.* 2022;15(1):14-20.

© 2024 Grashow R et al. *JAMA Neurology.*
