# Supplementary material for: Perceived Chronic Traumatic Encephalopathy and Suicidality in Former Professional Football Players
Source: JAMA Neurol. 2024 Sep 23;81(11):1130–9. doi: 10.1001/jamaneurol.2024.3083 (PMC11420824; doi:10.1001/jamaneurol.2024.3083)
Supplement: Supplement 2. — Data sharing statement [file jamaneurol-e243083-s002.pdf]

Data Sharing Statement

Data
Data available: No

Additional Information
Explanation for why data not available: Due to the high profile nature of the study participants, data are not available at this time.
